# Superficial cervical plexus block for management of herpes zoster neuralgia in the C3 dermatome: a case report

**DOI:** 10.1186/1752-1947-8-59

**Published:** 2014-02-19

**Authors:** Hye Young Shin, Doo Sik Kim, Sang Su Kim

**Affiliations:** 1 Department of Anesthesiology and Pain Medicine, Kosin University Gospel Hospital, 262 Gamcheon-ro, Seo-gu, Busan 602-702, Republic of Korea

**Keywords:** C3 dermatome, Herpes zoster, Neuralgia, Superficial cervical plexus block, Ultrasound

## Abstract

**Introduction:**

Herpes zoster is a well-known reactivating viral disease that gives rise to painful skin lesions. Although this vesicular rash heals up within a few weeks, pain sometimes continues, becoming postherpetic neuralgia. In the case of those at high risk of developing postherpetic neuralgia, early interventional pain management is generally recommended as a preventive measure. Pain specialists usually do not see patients face-to-face for chronic refractory pain until the stage of postherpetic neuralgia. However, active and aggressive management, including antiviral treatment, of herpetic neuralgia during the acute stage of herpes zoster promises better results. In this respect, superficial cervical plexus block can help patients, such as the case reported here, by relieving the pain of herpes zoster involving the C3 dermatome.

**Case presentation:**

A 65-year-old Korean man with severe pain in his left C3 dermatome due to herpes zoster was admitted to our hospital. His pain was so refractory to medication that he consulted our pain clinic for pain control. Due to the medication limitations imposed by his underlying diseases (hepatitis B, liver cirrhosis, atrial fibrillation, and asthma), early interventional therapy including stellate ganglion block was planned. In addition, because his painful C3 dermatome overlapped significantly with the superficial cervical plexus dermatome, ultrasound-guided superficial cervical plexus block was utilized for pain control of the intractable herpes zoster neuritis in his C3 dermatome. The result with respect to his sporadic neuralgia was satisfactory.

**Conclusions:**

We found superficial cervical plexus block to be an effective interventional procedure for pain management of herpes zoster, particularly at the C3-dermatomal level.

## Introduction

Herpes zoster (HZ) is a disease caused by reactivation of the varicella zoster virus (VZV) after initial presentation as chickenpox under conditions such as old age, malignancy, diabetes, and immunosuppressive therapy with/without steroids [1-3]. The median duration of illness is 7 to 10 days. Prodromal pain usually occurs unilaterally according to the involved dermatome. After 2 to 3 days of pain characterized by a burning or tingling sensation, erythematous maculopapular rashes appear and rapidly progress to vesicles. As the lesions dry, they become crusts, and typically peel away 3 to 5 days after their appearance. Recovery of normal skin in some cases takes several weeks. However, regardless of skin lesions, pain can persist and develop into postherpetic neuralgia (PHN). For this reason, it is important that early pain management be aggressive [2,4]. For patients at high risk of PHN, interventional management involving nerve block and other treatments together with medication is recommended [4]. Here we report the first known case of a patient with HZ neuritis involving the C3 dermatome who was effectively treated with superficial cervical plexus (SCP) block under ultrasound guidance.

## Case presentation

A 63-year-old Korean man with HZ presented at our hospital’s emergency room and was admitted to the dermatologic ward due to intractable medication-resistant pain. He had periodically received medical treatment for various conditions stemming from a history of atrial fibrillation, asthma, hepatitis B and liver cirrhosis. He had visited an otolaryngologic clinic for auricular pain that had arisen after a cold, although no ear abnormalities were found, 12 days before admission. Then, 2 days later, erythematous maculopapular rashes appeared in the painful area; subsequently, he was diagnosed with HZ and started on medications including antiviral agents. His skin lesions improved (excepting some vesicles) by degrees and crusts formed. Nevertheless, combined itching, stabbing and throbbing pain on the left of his neck and postauricular area did not improve. Just before he came to the emergency room, the throbbing pain had worsened considerably, manifesting itself at intervals of 2 to 3 hours despite medication. Finally, he was hospitalized due to refractory HZ neuralgia. Medication including nonsteroidal anti-inflammatory drugs, anticonvulsant (gabapentin, 600mg/day), antidepressants (amitriptyline, 10mg/day) and antibiotics was administered for alleviation of his symptoms, and he later consulted our pain clinic for pain control.

On physical examination at our pain clinic, he showed skin lesions with crusts mixed with some vesicles in the left C3 dermatome (that is, the left of his neck and postauricular area). The accompanying pain was continuously throbbing and burning in nature; shooting pain, additionally, was sporadic and intermittent. The intensity of his continuous pain registered six on the 11-point Numeric Rating Scale (NRS), and his intermittent pain, eight. It presented with allodynia and hyperalgesia. He also had pain-related sleep disturbance. The laboratory evaluation revealed a normal coagulation profile with a platelet count of more than 100,000/μL.

**Figure 1:**
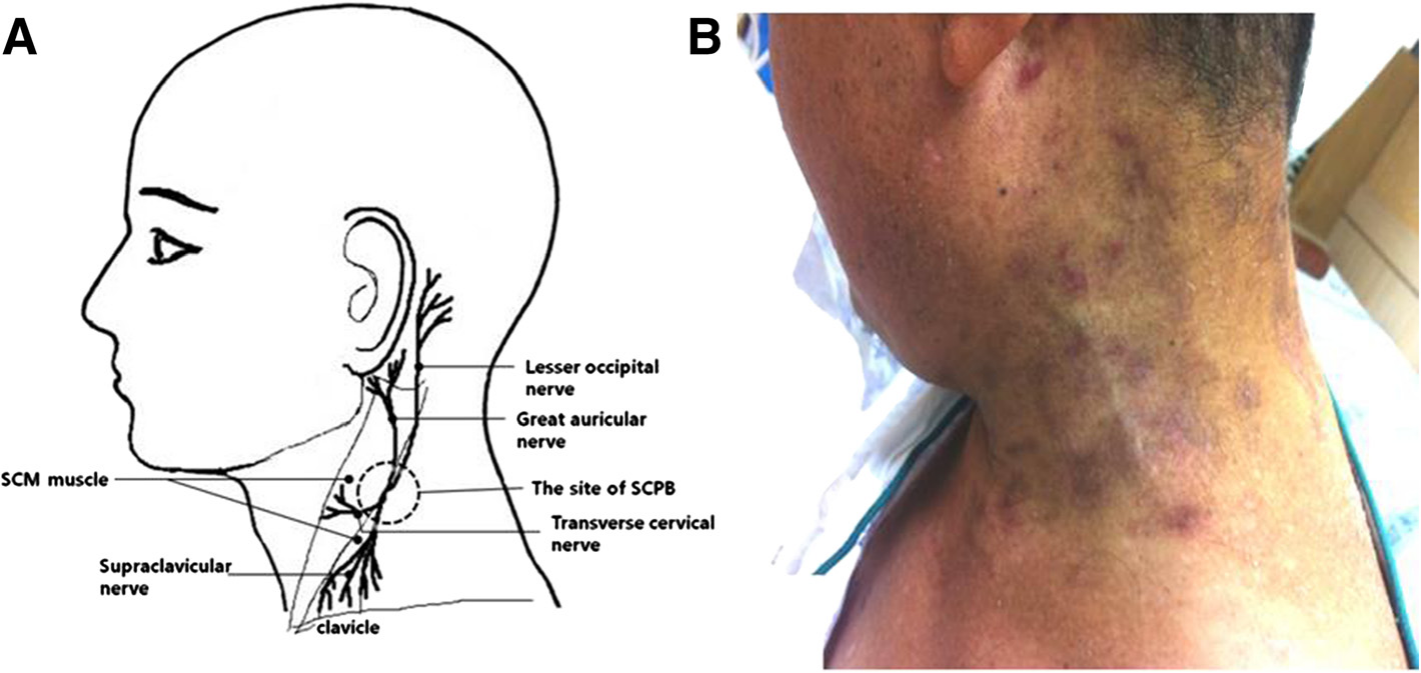
**Stellate ganglion block for effective treatment of neuralgia involving the C3 dermatome. (A)** An illustration of the four terminal branches of the superficial cervical plexus. **(B)** The patient’s skin lesion with the neuralgia due to herpes zoster. Abbreviations: SCM, sternocleidomastoid; SCPB, superficial cervical plexus block.

We performed stellate ganglion block (SGB) on the left of his neck, recommended augmented doses of gabapentin (to 1200mg/day) and amitriptyline (to 20mg/day), and prescribed EMLA® cream (a cream containing a eutectic mixture of local anesthetics: lidocaine and prilocaine). The following day, his pain was sufficiently alleviated to enable sleep. Routine laboratory findings included abnormal liver and renal function test data considered to have resulted from the patient’s underlying diseases. He was transferred to the Department of Internal Medicine and prescribed half doses of gabapentin (600mg/day) and amitriptyline (10mg/day). On the basis of his clinical condition, we administered intravenous patient-controlled analgesia (PCA) with fentanyl, and repeated the SGB four times at intervals of 2 or 3 days. Notwithstanding the gradual improvement of his symptoms, sporadic neuralgia, seemingly quite centralized in the dermatome of the SCP (Figure 1), still bothered him at dawn. In addition to the SGB, we performed ultrasound-guided SCP block with 6mL of 0.125% ropivacaine and 10mg of triamcinolone acetonide, after which his throbbing dawn pain disappeared. His NRS score fell to three through the course of the pain management. However, his allodynia and hyperalgesia did not quickly or significantly improve. About 10 days after starting the PCA, on the fifth day following the SCP block, he requested its discontinuance. The total numbers of SGB and SCP block applications were six and one, respectively, during his hospitalization.

## Discussion

HZ presenting as pain and characteristic skin lesions can become PHN although the skin lesions heal up. PHN manifests as continued intractable neuropathic pain in conjunction with abnormal sensation. High-risk factors for development of PHN include old age, severe acute pain, severe rash, prodromal symptoms of intense pain, and others. Moreover, PHN, like HZ itself with a heavy socioeconomic burden, reduces the patient’s quality of life. For all of these reasons, early aggressive management is strongly emphasized and recommended [2,5-8].

In diagnosing HZ, there are five symptoms to particularly watch out for: painful prodrome, unilateral dermatome involvement, vesiculopapular skin lesions, a history of rash in the same area, and skin lesion pain manifesting as allodynia [8]. Early diagnosis of HZ is extremely difficult at the prodromal stage lacking pathognomonic skin lesions; as such HZ is sometimes misdiagnosed as other diseases (for example appendicitis, myocardial infarction, renal colic, cholelithiasis, and so on) [4,6]. The commonly involved dermatomes range from T3 to L3. The fact that vesiculopapular skin lesions usually occur in these specific unilateral dermatomes is helpful to the diagnosis of HZ. If skin lesions are not definite, the highest sensitivity and highest specificity diagnostic tool for confirmation of VZV deoxyribonucleic acid (DNA) is polymerase chain reaction, which can be alternated with direct immunofluorescence assay [4]. In our present case, the patient with HZ involving the C3 dermatome, a less affected region, complained first only of a unilateral periauricular stinging pain, having no skin lesions, and thus could not easily be diagnosed with early stage HZ. Only once the C3 dermatomal rash was revealed could he be accurately diagnosed and treated for HZ with refractory pain.

For effective treatment of HZ, the antiviral agent must be prescribed within 48 to 72 hours to reduce the pain/skin lesion duration and PHN rate, particularly with elderly patients or patients who are immunodepressed. Even beyond 72 hours of rash onset, an antiviral agent, together with steroids, analgesics, antidepressants and so on, is recommended. The aforementioned high-risk group for development of PHN requires early and aggressive pain management with interventional therapy and medication. Interventions contain epidural block, sympathetic nervous system block, and nerve root or peripheral nerve block. Nerve blocks have shown effective pain control and even PHN prevention in some cases of acute HZ involving acute pain [2,4,7,9]. If the employed intervention is effective, it is repeated until pain is markedly improved; if not, then other interventions (that is, sympathetic neurolysis, spinal cord stimulation, peripheral nerve stimulation, pulsed radiofrequency lesioning or radiofrequency thermocoagulation of the dorsal root ganglion). Even though a number of methods, including those noted above, have been proposed, personalized or tailored treatment in consideration of specific effectiveness and prognosis is best [2,4,10]. Among the effective interventional therapies, SGB, a type of sympathetic nervous system blockade, can help to reduce HZ pain above the T3-dermatome level as a result of interaction between the sympathetic nervous system and the damaged nerve [2-4,10,11]. In the present case, the initial SGB procedure allowed our patient to sleep well at night, and was administered at least three times per week. Allodynia and hyperalgesia with sporadic throbbing pain localized in the C3 dermatome on the left of his neck remained, despite considerable overall alleviation of his pain. Regardless, his symptomatic area corresponded so completely with the SCP domain that a SCP block was planned. The SCP emerges from the anterior rami of the C1 to C4 dermatomes and leads to four terminal branches: the greater auricular nerve, the lesser occipital nerve, the transverse cervical nerve, and the supraclavicular nerve. The greater auricular nerve is the largest branch in the distribution of the anterior and posterior parts of the auricles; the lesser occipital nerve extends to the superior posterior neck and scalp behind the auricle; the transverse cervical nerve traverses the anterolateral territory of the neck from the sternum to the mandible; and the supraclavicular nerve separates into three branches supplying the cape of the neck and shoulder to the second intercostal level (Figure 1A).

We performed a SCP block at the level of the cricoid cartilage toward the hyperechoic fascia with reference to the posterior border of the sternocleidomastoid muscle, by the ultrasound-guided posterior in-plane approach using a linear high-frequency transducer and real-time assessment. At the target site, 6mL of 0.125% ropivacaine was injected with a 25G needle; it was enough for a successful blockage [12]. From the first day of the blockade, the sporadic throbbing and shooting pain completely disappeared; this outcome was highly satisfactory to the patient (Figure 1B).

## Conclusions

Whereas SGB is an interventional modality that is often employed, instances of its combination with SCP block in the treatment of HZ involving the C3 dermatome are almost unprecedented. Thus, we suggest that in cases where patients complain of periauricular pain without lesions or other apparent origin, there is a need to rule out the possibility of prodromal-phase HZ involving the C3 dermatome. Also, on the basis of the results of this case report, we recommend SCP block as a potentially very effective, minimally invasive and ultrasound-guidable treatment option for the control of intractable pain corresponding to the distribution of the SCP.

## Consent

Written informed consent was obtained from the patient for publication of this case report and accompanying images. A copy of the written consent is available for review by the Editor-in-Chief of this journal.

## Competing interests

The authors declare that they have no competing interests.

## Authors’ contributions

HS, DK and SK wrote, revised, read and approved the final manuscript. HS performed the ultrasound-guided superficial cervical plexus block.
